# Supplementary material for: Time-series transcriptome analysis identified differentially expressed genes in broiler chicken infected with mixed Eimeria species
Source: Front Genet. 2022 Aug 8;13:886781. doi: 10.3389/fgene.2022.886781 (PMC9393255; doi:10.3389/fgene.2022.886781)
Supplement: Supplementary file 2 [file DataSheet1.ZIP › 7dpi_GO.Gsea.1625106495923/GOBP_LIPID_PHOSPHORYLATION.html]

Details for gene set GOBP\_LIPID\_PHOSPHORYLATION[GSEA]

|  || Dataset | TMM\_7dpi\_gct\_format\_7dpi\_gct\_format.Class\_4dpi.cls #PC\_versus\_NC.Class\_4dpi.cls #PC\_versus\_NC\_repos |
| Phenotype | Class\_4dpi.cls#PC\_versus\_NC\_repos |
| Upregulated in class | 0 |
| GeneSet | GOBP\_LIPID\_PHOSPHORYLATION |
| Enrichment Score (ES) | -0.54398036 |
| Normalized Enrichment Score (NES) | -1.8228177 |
| Nominal p-value | 0.0 |
| FDR q-value | 0.10621474 |
| FWER p-Value | 0.899 |
Table: GSEA Results Summary

  

Fig 1: Enrichment plot: GOBP\_LIPID\_PHOSPHORYLATION      
 Profile of the Running ES Score & Positions of GeneSet Members on the Rank Ordered List

  

| SYMBOL | TITLE | RANK IN GENE LIST | RANK METRIC SCORE | RUNNING ES | CORE ENRICHMENT || 1 | SPHK1 | na | 171 | 1.089 | 0.0176 | No |
| 2 | PIK3C2B | na | 743 | 0.616 | -0.0121 | No |
| 3 | SOCS1 | na | 864 | 0.566 | -0.0056 | No |
| 4 | BPNT1 | na | 1352 | 0.417 | -0.0342 | No |
| 5 | INPP1 | na | 2880 | 0.141 | -0.1578 | No |
| 6 | SOCS4 | na | 2946 | 0.133 | -0.1593 | No |
| 7 | ITPKC | na | 3116 | 0.112 | -0.1702 | No |
| 8 | PIP5K1C | na | 3125 | 0.111 | -0.1676 | No |
| 9 | PIP5K1B | na | 3713 | 0.039 | -0.2156 | No |
| 10 | PI4K2A | na | 3872 | 0.019 | -0.2282 | No |
| 11 | DGKB | na | 4206 | -0.018 | -0.2555 | No |
| 12 | IP6K2 | na | 4374 | -0.038 | -0.2684 | No |
| 13 | AGK | na | 4571 | -0.058 | -0.2831 | No |
| 14 | DGKA | na | 5166 | -0.118 | -0.3293 | No |
| 15 | IP6K1 | na | 5426 | -0.141 | -0.3469 | No |
| 16 | PI4KB | na | 5680 | -0.167 | -0.3631 | No |
| 17 | PIP5K1A | na | 5711 | -0.171 | -0.3607 | No |
| 18 | PIP4K2B | na | 5735 | -0.173 | -0.3575 | No |
| 19 | IMPA1 | na | 5761 | -0.177 | -0.3544 | No |
| 20 | EFR3A | na | 6247 | -0.229 | -0.3883 | No |
| 21 | SOCS5 | na | 6883 | -0.295 | -0.4328 | No |
| 22 | PI4K2B | na | 7412 | -0.353 | -0.4666 | No |
| 23 | SOCS6 | na | 7508 | -0.362 | -0.4640 | No |
| 24 | DGKH | na | 7586 | -0.370 | -0.4596 | No |
| 25 | TTC7B | na | 8210 | -0.447 | -0.4986 | No |
| 26 | ITPKA | na | 8252 | -0.453 | -0.4888 | No |
| 27 | PIK3CG | na | 8478 | -0.481 | -0.4935 | No |
| 28 | IMPA2 | na | 9082 | -0.562 | -0.5275 | Yes |
| 29 | DGKZ | na | 9155 | -0.574 | -0.5167 | Yes |
| 30 | SOCS2 | na | 9342 | -0.605 | -0.5146 | Yes |
| 31 | CERK | na | 9446 | -0.623 | -0.5050 | Yes |
| 32 | DGKG | na | 9451 | -0.624 | -0.4870 | Yes |
| 33 | PIK3R2 | na | 9597 | -0.645 | -0.4803 | Yes |
| 34 | PIP4K2A | na | 9723 | -0.664 | -0.4713 | Yes |
| 35 | SLA2 | na | 9784 | -0.671 | -0.4567 | Yes |
| 36 | PI4KA | na | 9785 | -0.671 | -0.4370 | Yes |
| 37 | PIK3C2G | na | 10128 | -0.737 | -0.4440 | Yes |
| 38 | IPMK | na | 10143 | -0.741 | -0.4235 | Yes |
| 39 | PIK3CB | na | 10335 | -0.777 | -0.4168 | Yes |
| 40 | SMG1 | na | 10563 | -0.824 | -0.4116 | Yes |
| 41 | EFR3B | na | 10682 | -0.850 | -0.3966 | Yes |
| 42 | PIK3CD | na | 10707 | -0.855 | -0.3735 | Yes |
| 43 | FAM126A | na | 10717 | -0.858 | -0.3492 | Yes |
| 44 | PIK3CA | na | 10920 | -0.912 | -0.3394 | Yes |
| 45 | PIK3R6 | na | 10955 | -0.924 | -0.3152 | Yes |
| 46 | PIK3R3 | na | 11045 | -0.949 | -0.2948 | Yes |
| 47 | CISH | na | 11064 | -0.955 | -0.2684 | Yes |
| 48 | PIK3C3 | na | 11107 | -0.966 | -0.2436 | Yes |
| 49 | SOCS3 | na | 11181 | -0.994 | -0.2206 | Yes |
| 50 | PIK3C2A | na | 11243 | -1.015 | -0.1960 | Yes |
| 51 | PIK3R1 | na | 11265 | -1.023 | -0.1678 | Yes |
| 52 | DGKQ | na | 11364 | -1.064 | -0.1448 | Yes |
| 53 | DGKE | na | 11540 | -1.132 | -0.1263 | Yes |
| 54 | TTC7A | na | 11563 | -1.150 | -0.0945 | Yes |
| 55 | PIKFYVE | na | 11699 | -1.238 | -0.0696 | Yes |
| 56 | DGKK | na | 11898 | -1.495 | -0.0423 | Yes |
| 57 | DGKD | na | 11969 | -1.764 | 0.0034 | Yes |
Table: GSEA details [plain text format]

  

Fig 2: GOBP\_LIPID\_PHOSPHORYLATION      
 Blue-Pink O' Gram in the Space of the Analyzed GeneSet

  

Fig 3: GOBP\_LIPID\_PHOSPHORYLATION: Random ES distribution      
 Gene set null distribution of ES for **GOBP\_LIPID\_PHOSPHORYLATION**
